# Infective Endocarditis-Like Presentation of Felty Syndrome: A Case Report

**DOI:** 10.7759/cureus.20713

**Published:** 2021-12-26

**Authors:** Yoji Hoshina, Siale Teaupa, Derek Chang

**Affiliations:** 1 Department of Neurology, University of Utah, Salt Lake City, USA; 2 Department of Medicine, University of Utah, School of Medicine, Salt Lake City, USA

**Keywords:** differential diagnostic process, diferential diagnosis, physical diagnosis, rheumatology & autoimmune diseases, academic rheumatology

## Abstract

Felty syndrome (FS) and infective endocarditis (IE) can present with similar signs and symptoms. FS is a diagnosis of exclusion, which poses a challenge for the clinician since accurate diagnosis is required to treat this condition effectively.

A 52-year-old woman with a 15-year history of rheumatoid arthritis (RA) was admitted due to dyspnea and pain in the right ankle and left arm for two weeks. She was hemodynamically stable and afebrile. Physical examination revealed right ankle swelling and tenderness, left forearm tenderness, abdominal distension, and swan-neck finger deformities. Laboratory tests were notable for pancytopenia with a white blood cell (WBC) count of 2900 × 10^3^/μL (absolute neutrophil count (ANC) of 1800/μL). Rheumatoid factor and anti-cyclic citrullinated peptide tests were positive. Synovial fluid analysis of the right ankle showed no crystals or bacteria, and a WBC count of 192 × 10^3^/μL. Left upper extremity computed tomography (CT) revealed two abscesses, in the forearm and elbow, respectively. CT chest and abdomen revealed a wedge-shaped consolidation in the left upper lobe, multiple bilateral pulmonary nodules, and splenomegaly. Abdominal ultrasonography showed portal hypertension with no clear findings of cirrhosis. Blood cultures were negative. Transthoracic echocardiography (TTE) and transesophageal echocardiography showed no vegetation. Incision and drainage were performed for the right ankle swelling, and left forearm and elbow abscesses. Left forearm abscess culture revealed *Staphylococcus hemolyticus*. Transbronchial needle aspiration and culture of the left upper lobe lesion showed acute and chronic inflammation with no signs of malignancy or microbial growth. Repeat TTE and blood cultures were negative. Bone marrow biopsy and flow cytometry showed no evidence of large granular lymphocytic (LGL) leukemia. The patient was diagnosed with FS complicated by disseminated infections and pulmonary necrobiotic nodules. Empiric ceftriaxone and vancomycin were initiated. The patient was discharged after the resolution of her symptoms.

FS is a rare extra-articular presentation of RA with a triad of a > 10-year history of RA, neutropenia (ANC < 2000/μL), and splenomegaly. IE can also present with disseminated infections and splenomegaly. Repeat TTE and blood cultures were performed due to concerns regarding the high mortality rate of IE and the possibility of false-negative echocardiography results. LGL leukemia also presents with RA and neutropenia, which was deemed less likely in our patient based on unremarkable bone marrow biopsy and flow cytometry results.

FS is a rare condition. Therefore, it is important to keep its possibility in mind in the setting of RA while performing workup for the most likely conditions.

## Introduction

Felty syndrome (FS) is a rare extra-articular presentation of rheumatoid arthritis (RA) first reported in 1924 by Augustus Roi Felty [1]. It affects 1% of patients with RA with a triad of chronic RA, neutropenia (absolute neutrophil count (ANC) below 2000/μL), and splenomegaly [1,2]. Other clinical manifestations of FS include anemia, thrombocytopenia, idiopathic portal hypertension, and malignancy. It presents more commonly in Caucasian women who are usually diagnosed in the fifth to seventh decade of life and have a long-standing history of RA (10 or more years) until neutropenia is recognized [2]. The diagnosis of FS can be challenging since there is no specific diagnostic test, and the triad does not necessarily occur in all patients. In this report, we present a case of FS associated with necrobiotic lung nodules complicated with disseminated infection, mimicking infective endocarditis (IE).

## Case presentation

A 52-year-old Caucasian woman with a 15-year history of seropositive, erosive RA was admitted due to right ankle pain, left upper extremity pain, and dyspnea for two weeks. The patient also reported fatigue and night sweats but denied fever, chills, or cough. The patient had only been taking non-steroidal anti-inflammatory drugs (NSAIDs) for her arthritis. The patient had never been treated by a rheumatologist, nor been on any disease-modifying anti-rheumatic drug (DMARD) therapy or other immunosuppressive therapy, and expressed concern regarding the side effects of DMARDs. At the time of admission, she was also taking cefdinir and doxycycline which was prescribed at another hospital one week ago. Her medical history was notable for right knee septic arthritis 10 months prior, but she had no history of joint replacements. She had a 60 pack-year smoking history but did not have a history of excessive alcohol intake or illicit drug use, recent travel, or family history of autoimmune disease.

**Figure 1:**
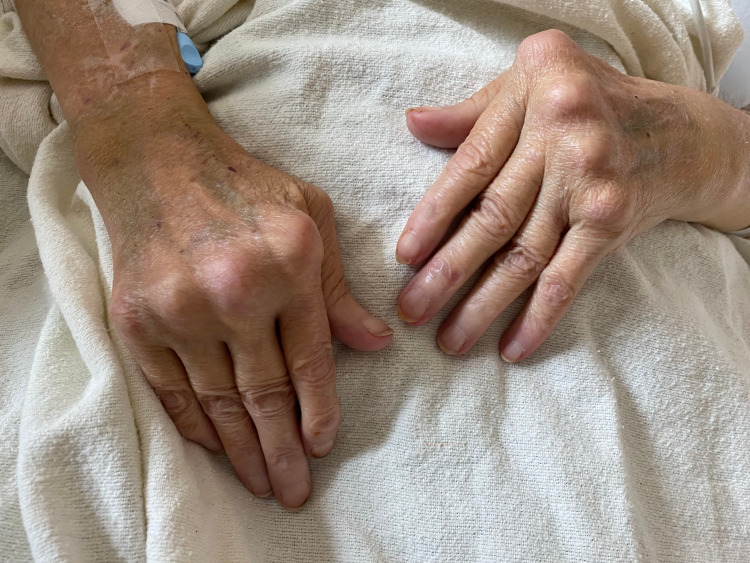
Ulnar deviation of metacarpophalangeal joints with swan-neck deformity of fingers.

She was hemodynamically stable and afebrile. Physical examination revealed right ankle swelling and tenderness, left forearm tenderness, and abdominal distension. On auscultation, breath sounds were diminished bilaterally. Her fingers showed characteristic deformities seen in RA (Figure 1).

**Figure 2:**
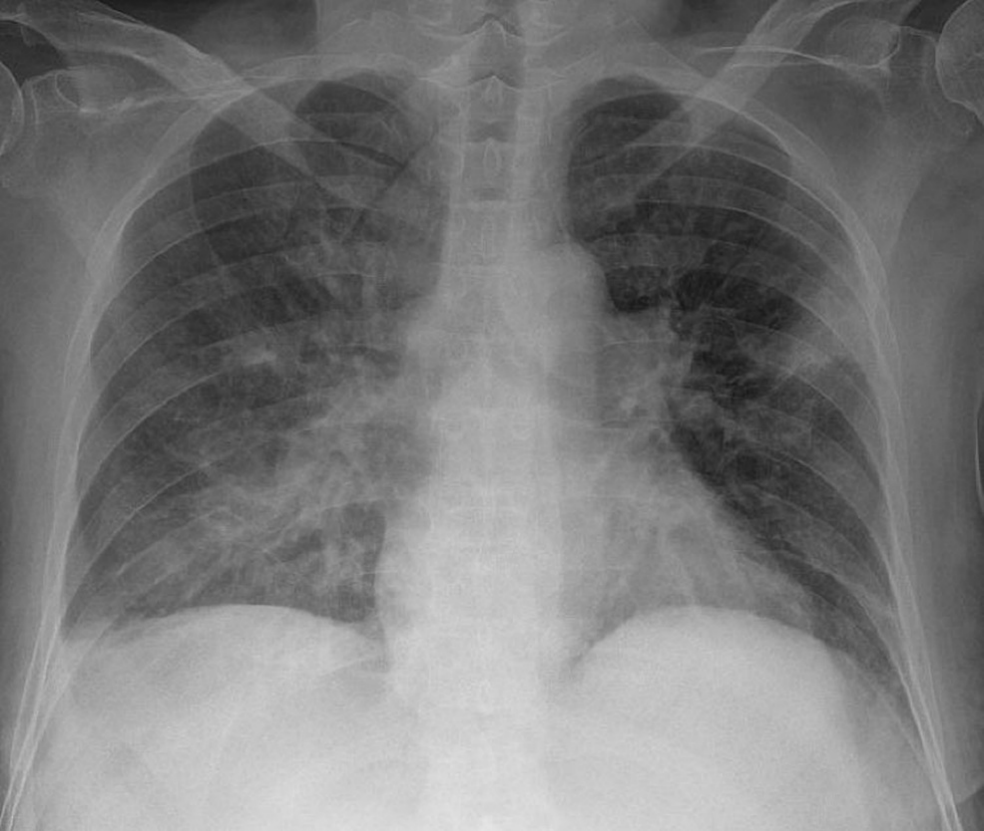
Presence of bilateral distribution of multifocal airspace opacities along with a small right pleural effusion.

Initial laboratory tests were notable for pancytopenia, with a white blood cell (WBC) count of 2900/μL (ANC of 1800/μL), hemoglobin of 8.4 g/dl, and platelet count of 123 × 10^3^/μL with no hemolysis on peripheral smear. Basic metabolic panel and liver function tests were unremarkable. C-reactive protein was 1.4 mg/L and erythrocyte sedimentation rate was 140 mm/hr. Rheumatoid factor and anti-cyclic citrullinated peptide tests were positive, with levels > 7150 IU/mL and > 200 units/mL, respectively. Chest radiograph revealed multifocal airspace opacities bilaterally with a small right pleural effusion (Figure 2). 

**Figure 3:**
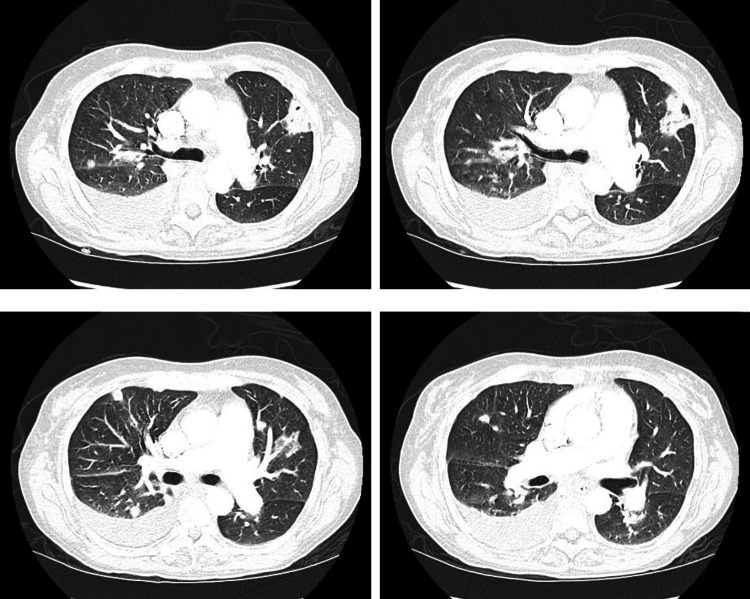
Wedge-shaped consolidation in the left upper lobe and multiple pulmonary nodules distributed randomly.

**Figure 4:**
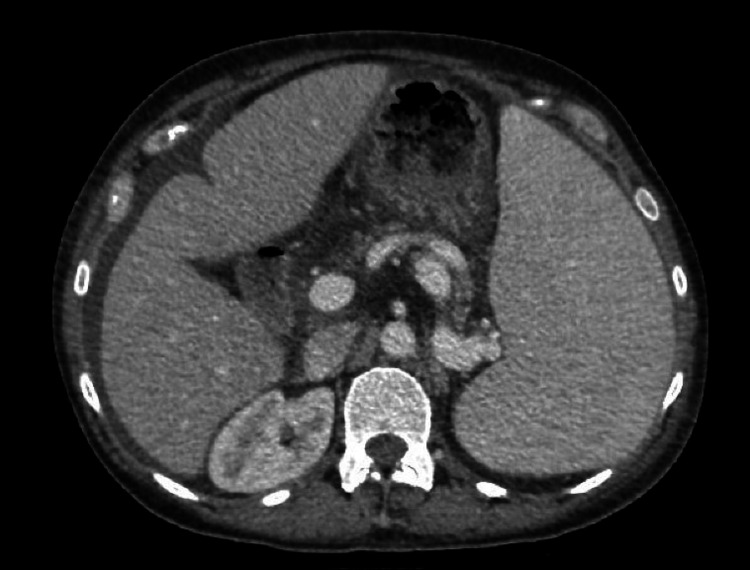
Enlarged spleen measuring 19.2 cm.

**Figure 5:**
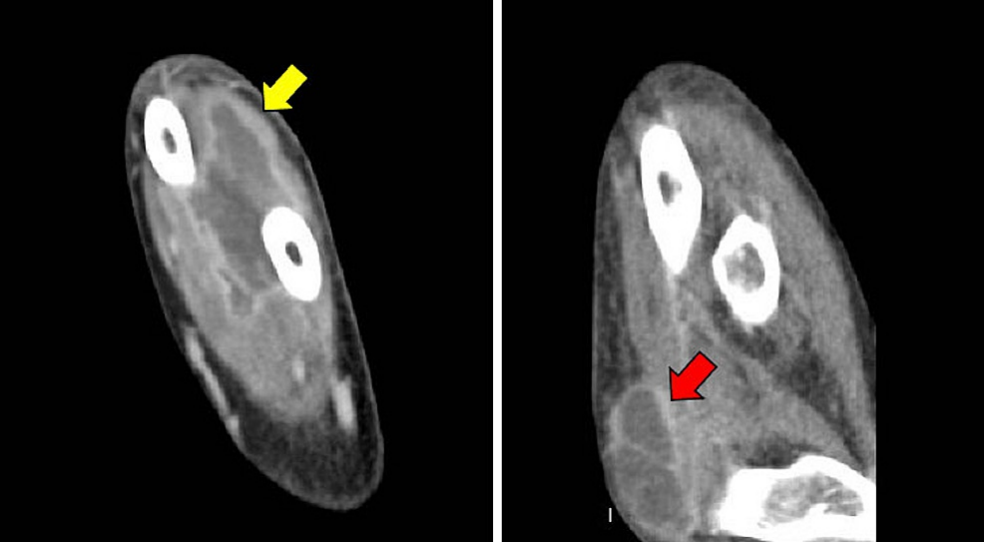
Deep space abscess (9.0 × 3.4 × 2.7 cm) in the mid and distal forearm (yellow arrow) and a 5.0 cm superficial abscess in the medial elbow (red arrow.)

Contrast-enhanced computed tomography (CT) of the chest revealed consolidation in the left upper lobe which appeared wedge-shaped, multiple pulmonary nodules scattered in a random distribution bilaterally, and moderate-sized right pleural effusion (Figure 3). Abdominal CT and ultrasonography revealed splenomegaly of 19.2 cm (Figure 4) and findings suggestive of portal hypertension without cirrhosis. Left upper extremity contrast-enhanced CT revealed a 9.0 × 3.4 × 2.7 cm deep space abscess in the mid-distal forearm between the radius and ulna and a 5.0 cm superficial abscess in the medial elbow without signs of osteomyelitis or joint effusions (Figure 5). Analysis of the synovial fluid obtained from the right ankle revealed a WBC count of 192 × 10^3^/μL. No crystals were identified, and gram staining was negative. Incision and drainage of the synovial fluid of the right ankle, and abscesses in the left forearm and elbow were performed. Gram staining of her left forearm abscess revealed gram-positive cocci.

The patient was started on empirical antibiotic therapy with vancomycin and ceftriaxone after collecting blood cultures. Transthoracic echocardiography (TTE) and transesophageal echocardiography showed no vegetation. Additional tests including hepatitis panel, antinuclear antibodies, human immunodeficiency virus antigen-antibody combination assay (fourth generation), QuantiFERON Gold test for tuberculosis, Aspergillus galactomannan antigen, Blastomyces antibody, Coccidiodes IgG/IgM, Histoplasmaantigen, and (1,3)-beta-D-glucan for invasive fungal infections were all negative. Transbronchial needle aspiration of the left upper lobe lesion showed acute and chronic inflammation with no signs of malignancy. Bone marrow biopsy showed normocellular and maturing trilineage hematopoiesis. Flow cytometry showed no evidence of a lymphoproliferative disorder. Forearm abscess culture grew Staphylococcus hemolyticus which was resistant only to penicillin. Cultures of blood, right ankle synovial fluid, left elbow abscess, and bronchoalveolar fluid were negative. Repeat blood cultures were negative. TTE was also repeated after 1 week to definitively rule out IE, given the risk of missed diagnosis, which again showed no vegetations. The patient was diagnosed with FS associated with pulmonary manifestation, likely necrobiotic lung nodules complicated by disseminated infections. She was treated with vancomycin and ceftriaxone for 4 weeks with subsequent resolution of her symptoms. After completion of antibiotic treatment, we initiated prednisone 0.5 mg/kg for FS. We planned to commence treatment with methotrexate once the patient was agreeable to initiating DMARDs.

## Discussion

We describe the case of a patient with RA who presented with characteristic features of FS complicated by disseminated infections and concomitant necrobiotic lung nodules. The final diagnosis was made after IE was ruled out. FS is a clinical diagnosis with no single diagnostic test. Although the presentation of FS can vary, neutropenia must be present for establishing the diagnosis [1]. In this case, the diagnosis of FS was particularly challenging since it presented alongside pulmonary manifestation, likely necrobiotic lung nodules, which is an extra-articular manifestation of RA. The nodule in necrobiotic lung nodules may be single or multiple, can multiply or regress independently, can be complicated with pleural effusion, and may also undergo cavitation [3]. Obliterative bronchiolitis (OB), which is another pulmonary manifestation seen in RA patients that can cause pulmonary nodules, was also considered [4]. In this case, the chest CT did not reveal mosaic attenuation, which is typical of OB. However, this mosaicism was reported in only 51.3% of the patients with OB [4]. Other differential diagnoses included drug-induced lung toxicity or Caplan syndrome, but the patient was not on any long-term medications, except NSAIDs, and had no previous exposure to coal dust. The findings of the transbronchial needle aspiration were unspecific for any conditions. The presence of these nodules in the setting of disseminated infection raised the possibility of septic emboli from IE, or one of the disseminated infection sites. Patients with FS are predisposed to recurrent infections. Skin and soft tissue infection or respiratory tract infection due to uncommon species need to be considered [1]. In this case, the patient also had a past medical history of right knee septic arthritis. 

IE is a potentially lethal condition that is often complicated by extracardiac manifestations including disseminated infection and splenomegaly [5]. Given the high mortality rate of IE especially in neutropenic patients, and the possibility of false-negative echocardiography results particularly at the early stage of the disease [6], we decided to repeat TTE and blood cultures. The negative results of repeat TTE, blood cultures, and culture from transbronchial needle aspiration limited the possibility of septic emboli from IE or disseminated infection. 

The only positive culture was of Staphylococcus hemolyticus, which was grown from the left elbow abscess culture. S. hemolyticus is a member of the coagulase-negative staphylococci [7], and part of the human skin flora; however, it is also an emerging pathogen causing nosocomial infections [7]. It can be challenging to determine whether the specimen is a contaminant or significant pathogen. Additionally, in this case, the patient was already on cefdinir and doxycycline for 1 week, which can cause a false-negative result for many gram-negative bacteria and methicillin-resistant staphylococcus aureus (MRSA) infection. We decided to continue ceftriaxone and vancomycin for a total of four weeks to cover both gram-negative organisms and MRSA, given her immunocompromised status due to FS.

Large granular lymphocytic (LGL) leukemia should also be considered in the presence of neutropenia, recurrent infections, and anemia, especially in a patient with a history of RA [8]. Peripheral blood smear and bone marrow biopsy help rule out LGL leukemia. In this case, the unremarkable bone marrow biopsy and flow cytometry results made the possibility of hematologic malignancy less likely. Furthermore, the patient had other characteristic findings of FS, namely, a ten or more-year history of RA, thrombocytopenia, splenomegaly, and idiopathic portal hypertension [2]. 

Our case highlights the diverse presentation of FS and the high susceptibility of FS patients to infections. Although FS is a rare condition, it is important to keep its possibility in mind in the setting of RA while performing workup for the most likely conditions.

## Conclusions

FS is a rare extra-articular presentation of RA with a triad of chronic RA, neutropenia, and splenomegaly. Additional clinical manifestations and complications of FS include anemia, thrombocytopenia, idiopathic portal hypertension, malignancy, and recurrent bacterial infections. It is a diagnosis of exclusion with no specific diagnostic test. The diagnosis of FS can be challenging, especially when the patient presents with additional complications.

In this case, the patient's FS was associated with a pulmonary manifestation, which is another extra-articular presentation seen in RA patients, and was complicated with disseminated infection. This clinical presentation mimicked that of infective endocarditis (IE) or septic emboli from other sites, which can be fatal. This case also highlights that patients with FS are more susceptible to infections. Skin and soft tissue infections or respiratory tract infections resulting from uncommon pathogenic species must be considered. Although FS is a rare condition, it is essential to keep its possibility in mind in the setting of RA while performing workup for the most likely conditions.
